# The odor of origin: kinship and geographical distance are reflected in the marking pheromone of male beewolves (*Philanthus triangulum *F., Hymenoptera, Crabronidae)

**DOI:** 10.1186/1472-6785-7-11

**Published:** 2007-10-10

**Authors:** Martin Kaltenpoth, Johannes Kroiss, Erhard Strohm

**Affiliations:** 1 University of Würzburg, Department for Animal Ecology and Tropical Biology, Am Hubland, 97074 Würzburg, Germany; 2 University of Regensburg, Department for Zoology, 93040 Regensburg, Germany

## Abstract

**Background:**

Pheromones play an important role for mate finding and courtship in many insects. In species where males are the signaling sex, females are expected to choose among potential mates with regard to the emitter's quality and/or genetic compatibility. One important aspect is the balance between negative and positive effects of in- vs. outbreeding. In the present study, we aimed to assess the potential of the territory marking pheromone of European beewolves as an indicator for genetic compatibility in the context of female choice.

**Results:**

We analyzed the sex pheromone composition of male European beewolves (*Philanthus triangulum *F., Hymenoptera, Crabronidae) from eight different locations across Central Europe (six in Germany, one in England, and one in Italy). The pheromone constitutes a complex blend of various long-chain hydrocarbons (alkanes, alkenes, alcohols, ketones, and a carbon acid). We demonstrate that pheromone composition differs significantly among distant populations (regional scale), among subpopulations (local scale) and between families within subpopulations. The differences in the pheromone blend are positively correlated with geographical distances as might be expected according to an isolation-by-distance model. On a local scale, family membership has a larger effect on pheromone composition than subpopulation affiliation, while the reverse is true for the regional scale.

**Conclusion:**

Our results show that male pheromones can contain information on both kinship and geographical origin that may be used by females to choose adaptively among potential mates on the basis of their genetic distance.

## Background

In many animals, sexual signals vary with the degree of kinship as well as with geographical distribution. This has been shown for numerous species with acoustical courtship signals [1-3], but also for several taxa with sex pheromones [4-8]. Previous studies on chemical signals, however, have focused on pheromones produced by females; evidence for geographical variation in male sex pheromones is largely lacking [but see [9-12]].

Since there is usually a conflict of interest between the sexes [13], male sex pheromones are expected to underlie completely different selective pressures than female pheromones [14,15]. Male sexual signals often enable females to choose adaptively among potential mates by providing information on species affiliation and mate quality [16-19]. If males vary in their ability to provide essential resources to the females [20,21] or in their parasite or disease load [22-24] and such differences in quality are indicated in the males' signals females could benefit directly by choosing a high-quality male. Females may also benefit indirectly, if offspring quality depends on the genetic background of the male. Several models have been proposed to explain female choice based on indirect benefits, the most prominent of these being the "good genes" model [25-27], and the model of the "best compatibility" [20,28,29].

The genetic compatibility of a mate depends, among other things, on the degree of kinship which ranges from strict inbreeding to extensive outbreeding, both of which have certain advantages [30] and disadvantages [31,32]. According to the model of optimal outbreeding, females should choose a mate of a certain genetic distance to balance negative effects of inbreeding and outbreeding [31,33,34].

Male European beewolves establish small territories in the vicinity of female nest aggregations, and territories do not contain any resources essential for beewolf females [35,36]. The males apply a marking pheromone from a cephalic gland (the postpharyngeal gland, PPG) onto plants within their territory and defend the territory against intruding males in combat flights without physical contact [35-40]. The marking pheromone of male European beewolves comprises a complex blend of up to 55 compounds [38,40] that might contain important cues for females to assess male quality and/or compatibility.

Behavioral observations provide clear evidence that the marking pheromone of beewolf males attracts receptive females to the males' territories [37]. Females approach territories in a zigzagging flight pattern from the downwind side, probably orienting towards the windborne pheromone [37]. Copulations usually occur within the males' territories [35-37] and seem to be under the control of females since they can easily repel unwanted males by virtue of their larger body size [37] or refuse copulation by bending their abdomen tip downwards (E. Strohm, pers. observation). Territories of different males are often aggregated, thereby constituting a lek situation in which the females have an ideal opportunity to compare among potential mates and choose the most suitable [35,37]. Since the copulation is not preceded by any kind of visual display, female choice appears to be, at least predominantly, based on information obtained from the male sex pheromone (E. Strohm, M. Kaltenpoth, J. Kroiss unpublished data). The amount and composition of the male PPG content have been shown to differ between families [41] and to vary with the age of the males [42].

Beewolves have good flying abilities and it is likely that individuals from different (sub)populations meet in the field. Thus, the discrimination between males belonging to different (sub)populations may be an important factor influencing female mate choice decisions. Using combined gas chromatography-mass spectrometry (GC-MS), we investigated whether the marking pheromone of male European beewolves varies between populations, between subpopulations, and among families within subpopulations in a way that might provide a basis for female choice. We compared the relative effects of population and family association on the composition of the pheromone and discuss the consequences for optimal mate choice.

## Results

### Pheromone amount and composition

Using coupled GC-MS, we found a total of 25 substances in the male sex pheromone: (*S*)-2,3-dihydrofarnesoic acid (DHFS hereafter); (*Z*)-9-octadecen-1-ol; (*Z*)-10-nonadecen-2-one; 1-octadecanol; heneicosane; "Unidentified substance 1" (unknown 1); "Unidentified substance 2"; docosane; (*Z*)-11-eicosen-1-ol; a mixture of (*Z*)-9-, (*Z*)-7-tricosene, and Δx, y-tricosadiene; 1-eicosanol; tricosane; a mixture of (*Z*)-9-, (*Z*)-7-tetracosene, and Δx, y-tetracosadiene (C24en); tetracosane (C24an); a mixture of (*Z*)-9-, (*Z*)-7-pentacosene, and Δx, y-pentacosadiene (C25en); pentacosane (C25an); a mixture of 7-, 11-, and 13-methyl pentacosane (m-C25an); (*Z*)-9-hexacosene (C26en); hexacosane (C26an); Δ-16-pentacosen-8-one (C25one); a mixture of (*Z*)-9-, (*Z*)-7-heptacosene, and Δx, y-heptacosadiene (C27en); heptacosane (C27an); octacosane (C28an); nonacosane (C29an); hentriacontane (C31an). Several peaks had to be combined for the analysis, because they were not always clearly separated by the GC-MS. This applies to (*Z*)-9-octadecen-1-ol and (Z)-10-nonadecen-2-one (C19enone hereafter), 1-octadecanol and heneicosane (C18anol), "Unidentified substance 2" and docosane (C22ane), (*Z*)-11-eicosen-1-ol and tricosenes/tricosadiene (C20enol), and 1-eicosanol and tricosane (C23ane). Thus, the 25 detected substances were reduced to 20 variables that were included in the analysis. This procedure is conservative with regard to the hypotheses tested.

The total amount of pheromone extracted from *P. triangulum *males varied between 101 and 2508 μg (mean ± SD = 655 ± 377 μg). In both data sets, the sampled populations differed significantly in their total amount of pheromone (data set 1: ANOVA, F_5,254 _= 6.66, p < 0.01; data set 2: ANOVA, F_4,128 _= 3.99, p < 0.01).

### Chemical dimorphism

The pheromone composition showed a distinct dimorphism [see [40] for a detailed description of the dimorphism]. The two morphs differ mainly in the relative proportions of pentacosene (mixture of isomers with (*Z*)-9-pentacosene as the main component) and heptacosene (mixture of isomers with (*Z*)-9-heptacosene as the main component), and they can be distinguished unambiguously by the relative amount of heptacosene, which shows a clearly bimodal distribution [40]. The morph with the high proportion of pentacosene (in the following called C_25_-type) was overall the more common type (79.1% of all males) compared to the one having approximately equal proportions of penta- and heptacosene (C_25_/C_27_-type in the following; 20.9% of all males) [see also [40]]. The frequency of the C_25_-type varied considerably between the sampled populations from 8.3 to 100.0% in the sampled populations (mean ± SD = 72.6 ± 26.3%). Since chance variations in the proportion of C_25_- and C_25_/C_27_-type males among families and among populations can greatly influence the outcome of statistical analyses on the chemical differentiation, all of the following analyses were performed on C_25_- and C_25_/C_27_-type individuals combined as well as on C_25_-type individuals only.

### Population differentiation

**Figure 1:**
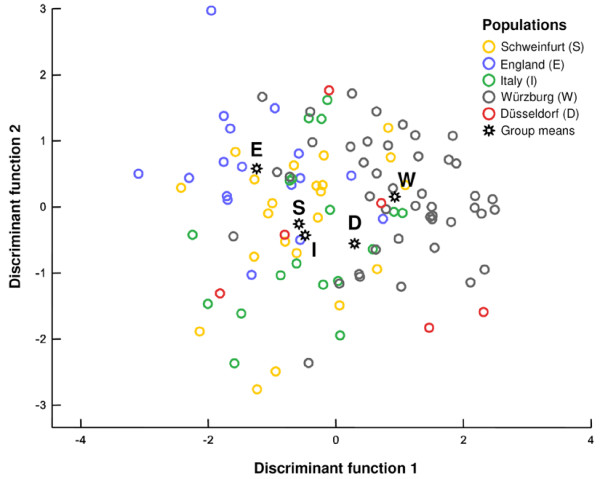
**Discriminant analysis of geographical variation of the sex-pheromone on the regional scale**. Despite some overlap, the populations are significantly separated (data set 2, five populations, C_25_-type only; see Table 1 and text for details).

**Figure 2:**
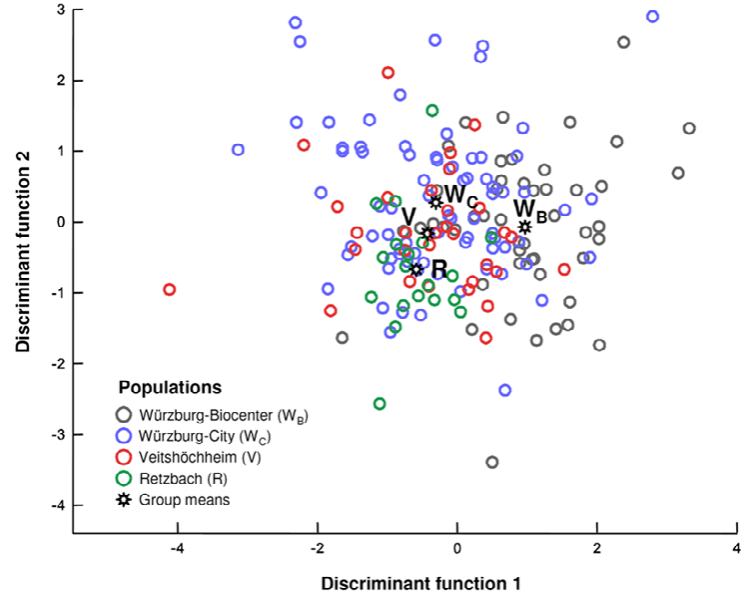
**Discriminant analysis of geographical variation of the sex-pheromone on the local scale**. Despite broad overlap, the populations are significantly separated (data set 1, four subpopulations, C_25_-type only; see Table 1 and text for details).

The analysis of geographical variation in the male beewolf sex pheromone was conducted with two data sets on two geographical scales. The first data set (data set 1) was focused on a local scale (subpopulation level), the second data set (data set 2) with emphasis on a regional scale (population level). Populations in both data sets and, thus, on two different spatial scales could be significantly separated by discriminant analyses (DAs) (Table 1, Fig. 1 and 2). This was irrespective of the inclusion or omission of the C_25_/C_27_-type in the analysis. Classification of DA revealed that 45.0 to 56.1% of males were correctly assigned to the populations, depending on the data set and inclusion or omission of the C_25_/C_27_-type (20.0 or 25.0% correct classifications would have been expected by chance). Despite the higher number of groups in the DA, the classification results were generally more accurate for the samples on the regional than on the local scale, indicating that the chemical distances were positively correlated with the geographical scale.

**Table 1:** Population and family differentiation by principal components and discriminant analyses

						**PCA**		**Discriminant analysis**					
**Scale**	**(Sub)Population(s)**	**Data set**	**Families**	**Type**	**n**	**Fact**.	**Expl.Var.(%)**	**Funct**.	**Wilk's-λ**	**χ**^2^	**df**	**p**	**Corr.class.(%)**
Regional	W. S. D. I. E	2	-	both	133	7	84.07	4	0.531	79.65	28	**0.000**	52.6
Regional	W. S. D. I. E	2	-	C_25_	107	7	83.79	4	0.485	72.40	28	**0.000**	56.1
Local	W_B_. W_C_. V. R	1	-	both	191	6	80.58	3	0.604	93.30	18	**0.000**	45.0
Local	W_B_. W_C_. V. R	1	-	C_25_	175	6	80.41	3	0.611	83.14	18	**0.000**	49.7

Family	W_B_	1	4	both	45	7	84.87	3	0.292	47.43	21	**0.001**	60.0
Family	W_B_	1	4	C_25_	44	7	86.09	3	0.240	53.44	21	**0.000**	75.0
Family	W_C_	1	7	C_25_	74	5	79.76	5	0.097	156.25	30	**0.000**	58.1
Family	W	2	4	C_25_	36	6	84.62	3	0.206	47.44	18	**0.000**	66.7
Family	V	1	3	both	28	4	81.03	2	0.557	13.73	8	0.089	60.7
Family	V	1	3	C_25_	24	4	77.46	2	0.614	9.51	8	0.301	62.5
Family	S	1	3	both	28	4	81.07	2	0.116	50.62	8	**0.000**	89.3
Family	S	2	3	both	20	3	66.36	2	0.358	16.45	6	**0.012**	60.0

"Type" indicates, whether only the C_25_-type or both C_25_- and C_25_/C_27_-type were included in the analysis. For PCAs, the number of factors as well as the cumulative explained variance is given. For DAs, the number of functions, Wilk's-λ, χ^2^, degrees of freedom, p-value, and the percentage of correct classifications by DA are given. (Population abbreviations: W: Würzburg, Germany; W_B_: Würzburg, Biocenter, Germany; W_C_: Würzburg, City, Germany; V: Veitshöchheim, Germany; R: Retzbach, Germany; D: Düsseldorf, Germany; I: Vizzola Ticino, Italy; E: Puttenham, UK).

### Family differentiation

**Figure 3:**
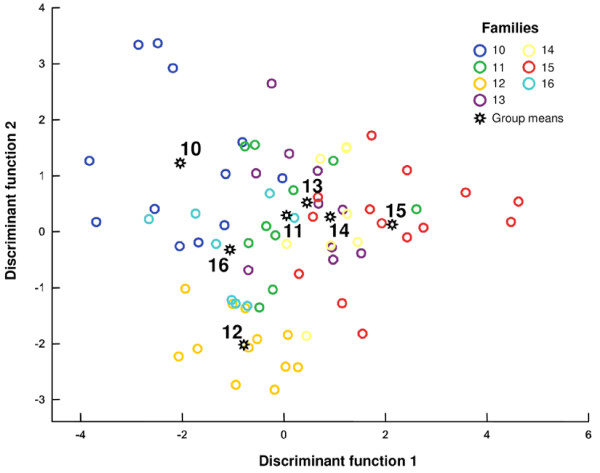
**Discriminant analysis of the variation of the sex-pheromone on the family level**. Despite some overlap, the families are significantly separated (data set 1, one population: Würzburg City, C_25_-type only; see Table 1 and text for details).

Families within populations could be significantly separated in six out of eight populations by DAs (Table 1, Fig. 3). Individual males were correctly classified in 58.1 to 100.0% of cases (14.3 to 50.0% correct classifications would have been expected by chance). Although DAs were not always significant, the overall classifications of families within populations were more accurate than classifications between populations even if different numbers of groups in the DA are taken into account. Thus, males belonging to different populations and males belonging to different families within a population can be separated from each other on the basis of quantitative differences in some of the pheromone compounds.

### Relative effects of family and population affiliation on pheromone composition

To assess the relative effects of family and population affiliation on the pheromone composition of male European beewolves, we conducted a multivariate nested ANOVA on the Aitchison-transformed relative peak areas with family membership as a nested factor within populations. Both family and population affiliation had significant effects on the pheromone composition in each dataset, regardless of the omission or inclusion of C_25_/C_27_-type males in the analysis (Table 2). Together, family and population affiliation explained between 11.4 and 90.8% of the variance in peak areas. The distribution of η^2^-values did not deviate significantly from a normal distribution for any of the analyses (Kolmogorov-Smirnov tests: Z ≤ 1.16, p ≥ 0.135 for all tests).

**Table 2:** Relative effects of family and (sub)population affiliation on pheromone composition in male European beewolves

	**Data set 1: Local C_25_- and C_25_/C_27_-type**		**Data set 1: Local C_25_-type only**		**Data set 2: Regional C_25_- and C_25_/C_27_-type**		**Data set 2: Regional C_25_-type only**	
**Peak**	**η^2^(Pop)**	**η^2^(Fam)**	**η^2^(Pop)**	**η^2^(Fam)**	**η^2^(Pop)**	**η^2^(Fam)**	**η^2^(Pop)**	**η^2^(Fam)**
DHFS	0.024	0.110	0.010	0.109	0.041	0.118	0.084	0.101
C19enone	0.089	0.243	0.086	0.250	0.439	0.060	0.462	0.105
C18anol	0.118	0.166	0.133	0.168	0.268	0.162	0.188	0.191
unknown1	0.055	0.074	0.033	0.081	0.273	0.070	0.207	0.076
C22ane	0.024	0.302	0.025	0.337	0.053	0.200	0.040	0.263
C20enol	0.088	0.063	0.083	0.072	0.137	0.085	0.152	0.079
C23ane	0.036	0.079	0.056	0.083	0.336	0.190	0.308	0.244
C24ene	0.044	0.354	0.012	0.376	0.069	0.185	0.162	0.139
C24ane	0.085	0.200	0.060	0.200	0.116	0.033	0.083	0.036
C25ene	0.001	0.190	0.016	0.217	0.062	0.156	0.126	0.154
C25ane	0.062	0.165	0.026	0.136	0.161	0.071	0.081	0.079
mC25ene	0.152	0.327	0.119	0.330	0.174	0.094	0.232	0.098
C26ene	0.001	0.182	0.107	0.301	0.284	0.072	0.307	0.096
C26ane	0.225	0.247	0.209	0.230	0.146	0.056	0.093	0.045
C25enone	0.012	0.239	0.012	0.243	0.222	0.289	0.397	0.511
C27ene	0.014	0.188	0.105	0.276	0.255	0.074	0.178	0.037
C27ane	0.205	0.388	0.191	0.397	0.133	0.141	0.091	0.083
C28ane	0.074	0.141	0.066	0.119	0.046	0.085	0.051	0.080
C29ane	0.080	0.166	0.045	0.165	0.219	0.140	0.168	0.080
C31ane	0.076	0.135	0.068	0.135	0.270	0.197	0.285	0.216

**MANOVA results:**								
**Pillai's trace**	**0.944**	**3.582**	**0.988**	**3.703**	**2.029**	**2.414**	**2.301**	**2.618**
**F**	**5.137**	**2.994**	**5.366**	**3.019**	**3.501**	**1.869**	**3.184**	**1.897**
**p**	**0.000**	**0.000**	**0.000**	**0.000**	**0.000**	**0.000**	**0.000**	**0.000**

Proportions of variance explained by family and population membership in nested MANOVAs are estimated for each peak by partial η^2^-values. MANOVA results are given for each data set, including and excluding C_25_/C_27_-type males, respectively (see text for peak abbreviations).

On a local scale (data set 1), family membership explained a significantly higher proportion of the variance in pheromone composition than subpopulation affiliation (paired t-tests, C_25_- and C_25_/C_27_-type: t_19 _= -6.22, p < 0.001; C_25_-type only: t_19 _= -6.17, p < 0.001). On a regional scale (data set 2), however, this effect was reversed, with population affiliation explaining more of the variance, although this effect was only significant when both C_25_- and C_25_/C_27_-type males were included (paired t-tests, C_25_- and C_25_/C_27_-type: t_19 _= 2.11, p = 0.048; C_25_-type only: t_19 _= 1.84, p = 0.081).

### Correlation between geographical and chemical distance

**Figure 4:**
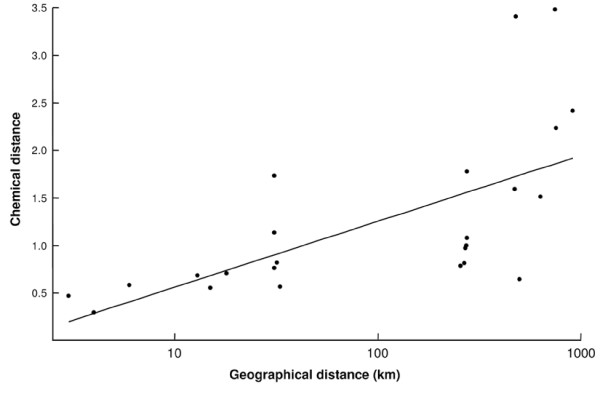
**Correlation between pairwise geographic and chemical distances of populations of *P. triangulum *males**. The trend line was obtained by linear regression in order to visualize the association (data sets 1 and 2; C_25_-type only; normalization: Würzburg – Düsseldorf; Mantel-test: r = 0.630, p = 0.013; see also Table 4 and text for details).

To test for a correlation between the matrices of geographical and chemical distances of the populations we performed a Mantel test. We detected a strong correlation between geographical and chemical distance for data set 1 and the normalized combination of both datasets irrespective of which combination of populations was used as a reference for normalization and independent of the inclusion or omission of the C_25_/C_27_-type in the analysis (Tables 3 and 4, Fig. 4). A Mantel test restricted to data set 2 revealed no significant correlation.

**Table 3:** Correlation between geographic and chemical distances of populations of *P. triangulum*

					**Mantel test**	
**Data set**	**Scale**	**Populations**	**Type**	**Normalization**	**r^2^**	**p**
1	Local	W_B_, W_C_, V, R, S, D	both	-	**0.854**	**0.003**
1	Local	W_B_, W_C_, V, R, S, D	C_25_	-	**0.584**	**0.004**

2	Regional	W, S, D, I, E	both	-	0.092	0.254
2	Regional	W, S, D, I, E	C_25_	-	0.074	0.319

1 and 2	Local+Regional	W, W_B_, W_C_, V, R, S, D, I, E	both	S-W	**0.468**	**0.008**
1 and 2	Local+Regional	W, W_B_, W_C_, V, R, S, D, I, E	both	D-W	**0.608**	**0.003**
1 and 2	Local+Regional	W, W_B_, W_C_, V, R, S, D, I, E	both	S-D	**0.581**	**0.004**
1 and 2	Local+Regional	W, W_B_, W_C_, V, R, S, D, I, E	C_25_	S-W	**0.253**	**0.016**
1 and 2	Local+Regional	W, W_B_, W_C_, V, R, S, D, I, E	C_25_	D-W	**0.397**	**0.013**
1 and 2	Local+Regional	W, W_B_, W_C_, V, R, S, D, I, E	C_25_	S-D	**0.362**	**0.005**

Given are coefficients of determination (r^2^) and p-values of Mantel tests. The column "type" indicates, whether only the C_25_-type or both C_25_- and C_25_/C_27_-type were included in the analysis. Data sets 1 and 2 were normalized to Würzburg – Schweinfurt (W-S), Würzburg – Düsseldorf (W-D), or Schweinfurt – Düsseldorf (S-D). For population abbreviations see Table 1. P-values < 0.05 are given in bold.

**Table 4:** Geographical and chemical distances between the nine sampled (sub)populations of *P. triangulum*

	**W**	**W_B_**	**W_C_**	**V**	**R**	**S**	**D**	**I**	**E**
**W**	-	*NA*	4	6	18	31	274	472	746
**W_B_**	*NA*	-	4	6	18	31	274	472	746
**W_C_**	*NA*	*0.29*	-	3	15	32	270	473	743
**V**	*NA*	*0.58*	*0.47*	-	13	33	267	474	740
**R**	*NA*	*0.70*	*0.55*	*0.68*	-	31	256	485	732
**S**	*1.73*	*0.76*	*0.82*	*0.57*	*1.13*	-	276	501	758
**D**	*1.00*	*1.00*	*0.97*	*0.81*	*0.79*	*1.08*	-	635	479
**I**	*1.59*	*NA*	*NA*	*NA*	*NA*	*0.64*	*1.51*	-	911
**E**	*3.48*	*NA*	*NA*	*NA*	*NA*	*2.23*	*3.40*	*2.41*	-

Chemical distances (normalized to Würzburg – Düsseldorf) are displayed *in italics *in the lower left half of the table, geographical distances in the upper right half of the table. For population abbreviations see Table 1.

## Discussion

In the present study, we investigated inter-individual differences in the pheromone composition of male European beewolves on three different levels: between families, among subpopulations on a local scale, and among geographically distant populations on a regional scale. Our results show that there are significant differences in pheromone composition on all three levels and that the chemical distance between populations is correlated with the geographical distance.

Although local subpopulations as well as geographically distant populations of beewolves could be separated on the basis of the male sex pheromone, only 45 – 56% of the individual males were classified correctly by the discriminant analyses, and chemical profiles of different populations overlapped considerably. However, the existing differences might be sufficient for females to reduce the incidence of outbreeding, especially because the sensitivity of female chemoreceptors and the discriminatory ability of their central nervous system may exceed that of our analytical methods by several orders of magnitude [43,44]. Furthermore, our data provide only a lower boundary for the actual effect of geographical origin on the pheromone composition, since all the animals were reared under identical conditions in the laboratory. In the field, developmental conditions certainly vary between (sub)populations. Differences in environmental factors during larval development have been shown to affect the pheromone composition of male beewolves (K. Roeser-Mueller, M. Kaltenpoth & E. Strohm, unpubl. data). Therefore, actual differences between populations in the field may be much larger than those observed under controlled conditions in the laboratory and might allow females a better discrimination of males from different (sub)populations.

Since beewolves have good flying abilities and are pioneer species that frequently colonize new habitats [45], females are likely to encounter males from other local subpopulations in the field (whereas it is unlikely that they encounter individuals from distant populations). Thus, female beewolves may use the information contained in the male pheromone to avoid outbreeding depression. Deleterious effects of extensive outbreeding have been demonstrated in many recent studies [46-48], and several hypotheses have been proposed to explain why outbreeding depression occurs [e.g. break-up of coadapted gene complexes, disruption of epistatic interactions, loss of local adaptations, dispersal hazards, and risk of parasite infection; see [31,32]].

Within populations, beewolf male pheromones differ significantly among families [this study and [41]]. The family-related differences may enable females to reduce the chances of mating with close kin and thereby avoid inbreeding depression, which is likely to impose especially high costs on beewolves. Kin recognition in animals is generally mediated by one of three mechanisms: phenotype matching, recognition of genetically compatible mates, or imprinting or learning of the individuals that occur in the same nest or birth place [32,49,50]. The mechanisms by which female beewolves could distinguish between kin and non-kin are unclear [for discussion see [41]]. The discrimination of males from different populations may be possible for females by sampling pheromones from different males in a lek and avoiding individuals with a pheromone blend that differs markedly from the population mean. Further studies are necessary to elucidate the mechanisms of kin- and population-recognition in European beewolves.

The multivariate nested ANOVAs indicate that pheromone composition is affected more strongly by family than subpopulation affiliation on a local scale, whereas the effect of population affiliation on a regional level is larger than the family effect (Table 2). These results are consistent with the positive correlation between chemical and geographical distance (Fig. 4) and suggest that (1) the subpopulations sampled on the local scale may be connected by a relatively frequent interchange of individuals and might, thus, represent a single population, and (2) small differences in the chemical profile between local subpopulations may add up on a regional scale according to an isolation-by-distance model [51]. Interestingly, the family effect exceeds the local subpopulation effect, making family differentiation potentially easier for beewolf females than subpopulation discrimination.

Generally, conspecific populations differ genetically only if the gene flow is sufficiently counterbalanced by the divergent forces of genetic drift or natural selection [52]. The reasons for the geographical differentiation in pheromone composition of male European beewolves are not yet known. However, selection pressures might vary among populations and may account for the observed differences. Males in colder regions, for example, may be selected for a greater abundance of pheromone substances with high volatility compared to males from warmer regions. Alternatively, female preferences for pheromone characteristics may vary among populations and cause a divergence of the pheromone composition at different localities.

## Conclusion

Using GC-MS, we were able to detect differences in the sex pheromone composition of male European beewolves between families as well as among (sub)populations on both a regional and a local scale, with pheromone differentiation being significantly correlated with geographical distance. If female beewolves use this information on kinship and geographical origin contained in the male sex pheromone, they may be able to choose adaptively among potential mates according to the model of optimal outbreeding, thus, avoiding the deleterious effects of both in- and outbreeding by choosing a mate of intermediate genetic distance [31,53]. Studies considering both in- and outbreeding avoidance in an integrated model of "optimal outbreeding" are scarce [but see [54-56]]. The European beewolf constitutes an interesting model system to test for optimal outbreeding in a species with a complex male sex pheromone, and further studies may show whether females indeed use the male pheromone to avoid in- and outbreeding.

## Methods

### Insects and sampling

In 2004, four subpopulations with distances ranging from 3 to 18 km (median = 10 km) were sampled (data set 1: local scale; subpopulations: Würzburg, Biocenter, Germany, (49°46'47''N, 09°58'11''E), Würzburg, City, Germany, (49°47'56''N, 09°55'38''E), Veitshöchheim, Germany, (49°48'20''N, 09°53'22''E), Retzbach, Germany, (49°54'55''N, 09°49'16''E)). Additionally, specimens from two distant populations in Germany were sampled to allow the combination with data set 2 for the analysis of the association between chemical and geographical distance (Schweinfurt, Germany, (50°03'00''N, 10°14'00''E), and Düsseldorf, Germany, (51°11'13''N, 6°48'09''E)). The distant populations were not included in the analysis of the chemical differentiation between the subpopulations. In 2005, we were able to sample five populations with distances ranging from 31 to 911 km (median = 490 km) (data set 2: regional scale; populations: Würzburg, Germany, (49°46'47''N, 09°58'11''E), Schweinfurt, Germany, (50°03'00''N, 10°14'00''E), Düsseldorf, Germany, (51°11'13''N, 6°48'09''E), Vizzola Ticino near Milano, Italy, (45°37'35''N, 08°42'14''E), and Puttenham near London, UK, (51°13'20"N, 0°40'02"W)).

Female European beewolves were collected at each of the locations given above. They were transferred to laboratory cages at the University of Würzburg and reared after Strohm [36]. Cocoons with larvae of the F1 generation were placed individually in Eppendorf^® ^tubes and kept in boxes with moist sand at 10°C for four to nine months of overwintering. Cocoons were then transferred to warm conditions (cycles of 12 hours at 25°C and 12 hours at 22°C) and adult beewolves emerged four to six weeks later. Emerging males were marked individually with up to three spots of acrylic paint on the dorsal side of the thorax and were allowed to fly in a climate chamber (2.5 × 1.8 × 2.1 m in size) with 12 h light/dark cycles at 25°C/20°C and provided with honey *ad libitum*. Since very young males have been shown to considerably differ in amount and composition of the pheromone [42] males were all caught at an age of 12–17 days and kept in small polystyrol vials (height: 80 mm; diameter: 35 mm) with moist sand and a drop of honey for two days to allow the pheromone glands to be replenished. After anesthetizing the males with CO_2_, they were killed by freezing and kept frozen (at -20°C) until extraction of the pheromone and GC-MS analysis.

Overall, 393 males were used for the analysis (Data set 1: Würzburg, Biocenter, Germany: 54, Würzburg, City, Germany: 76, Veitshöchheim, Germany: 35, Retzbach, Germany: 26, Schweinfurt, Germany: 57, and Düsseldorf, Germany: 12; Data set 2: Würzburg, Germany: 46, Schweinfurt, Germany: 26, Düsseldorf, Germany: 8, Vizzola Ticino, Italy: 24, and Puttenham, UK: 29).

### Gas chromatography – mass spectrometry

Frozen males were decapitated and their heads were cut at both sides to open up the postpharyngeal gland, which is the storage organ of the male sex pheromone [40,57]. Heads were placed individually in glass vials (1.5 ml), and 20 μl of a 1 g/l solution of octadecane in hexane (equivalent to a final amount of 20 μg of octadecane) was added as an internal standard to each vial to allow quantification of the pheromone. The heads were then submerged in approximately 1 ml distilled hexane and chemicals were extracted for four hours.

After extraction, samples were analyzed immediately by coupled capillary gas chromatography-mass spectrometry (GC-MS) with an Agilent 6890N Series gas chromatograph (Agilent Technologies, Böblingen, Germany) coupled to an Agilent 5973 inert mass selective detector. The two data sets were run on the same GC-MS device, but with different capillary columns and slightly different temperature programs. GC-MS set-up 1 (data set 1): The GC was equipped with a HP-5 fused silica capillary column (J&W, 30 m × 0.32 mm ID; df = 0.25 μm; temperature program: from 60°C to 300°C at 5°C/min, held constant for 1 min at 60°C and for 10 min at 300°C). GC-MS set-up 2 (data set 2): The GC was equipped with a RH-5ms+ fused silica capillary column (J&W, 30 m × 0.25 mm ID; df = 0.25 μm; temperature program: from 120°C to 300°C at 3°C/min, held constant for 1 min at 120°C and for 1 min at 300°C).

Helium was used as the carrier gas with a constant flow of 1 ml/min. A split/splitless injector was used (250°C) with the purge valve opened after 60 sec. The electron impact mass spectra (EI-MS) were recorded with an ionization voltage of 70 eV, a source temperature of 230°C and an interface temperature of 315°C. Since preliminary analyses had revealed that the total amount of chemicals in the sample has an effect on the detection and quantification of certain components, samples in which the pheromone concentration was either too high or too low were rerun after adjusting the pheromone concentration by addition or evaporation of hexane.

### Statistical analysis

#### Pheromone amount and composition

In the pheromone extracts, 25 components could be reliably detected in all samples, and their peaks were manually integrated with MSD ChemStation software (Agilent Technologies). The substances were identified by comparison of mass spectra and retention times with earlier analyses [38,40]. Not all substances described as components of the pheromone by Kroiss *et al*. [40] could be detected due to the low concentrations of the pheromone extracted from single males. Using the octadecane peak as an internal standard, the total amount of pheromone was calculated and then log_10_-transformed to obtain normally distributed data for statistical analysis. The log_10_-transformed absolute amounts of pheromone were compared among populations by ANOVAs. SPSS 13.0 software was used for the calculations. The relative amounts of the 20 pheromone components were calculated (peak area/total peak area). Because the relative amounts constitute compositional data, they were transformed to logcontrasts prior to analysis [58].

#### Chemical dimorphism

A histogram with the Aitchison-transformed proportion of heptacosene revealed a clearly bimodal distribution without any overlap and, thus, allowed us to unambiguously assign males to the two different morphs (value < 0.55: C_25_-type; value ≥ 0.55: C_25_/C_27_-type; see [40] for a detailed description of the dimorphism, figures showing chromatograms of both morphs and a histogram with the frequency distribution of the proportion of heptacosene in a population of 45 males). Chance variations in the proportion of C_25_- and C_25_/C_27_-type males among families and among populations can greatly influence the outcome of statistical analyses on the chemical differentiation. Therefore, all of the analyses were performed on C_25_- and C_25_/C_27_-type individuals combined as well as on C_25_-type individuals only. Thus, by excluding the C_25_/C_27_-type males from the analysis, we could make sure that statistical differences between populations or families are due to the overall chemical profile rather than just the frequency of the two distinct chemical morphs. The sample size of C_25_/C_27_-type males was too small for a reasonable analysis excluding the C_25_-type individuals.

#### Population differentiation

The number of describing variables was reduced by principal components analyses (PCA, Aitchison-transformed relative amounts of pheromone components as variables, varimax rotation, factor extraction: eigenvalues > 0.8). The extracted PCA factors were used for DAs to test whether males of different populations can be separated based on their pheromone profiles. The number of PCA factors used for the DAs was restricted to a maximum of N/6 (N = total number of males in the analysis) to avoid an excess of variables that may increase the risk of false-positive results. This procedure is conservative with regard to the hypotheses tested. A PCA and a DA was conducted for each of the two data sets, respectively. For PCA and DA, data set 1 was restricted to the local scale, including only subpopulations in close spatial vicinity (Würzburg, Biocenter, Würzburg, City, Veitshöchheim, Retzbach: maximum distance: 18 km) to exclude regional effects on the outcome of the DA.

#### Family differentiation

To determine whether families within populations can be separated on the basis of the chemical profile, PCA and DA were conducted as described above for each population for which at least three families with five or more brothers were available. SPSS 13.0 software was used for the principal components and discriminant analyses.

#### Relative effects of family and population affiliation on pheromone composition

We conducted a multivariate nested ANOVA on the Aitchison-transformed relative peak areas with family membership as a nested factor within populations to assess the relative effects of family and population affiliation on the pheromone composition. For each data set, two ANOVAs were computed, one including both C_25_- and C_25_/C_27_-type males and the other one with C_25_-type individuals only. For every pheromone peak, the proportion of variance explained by the two factors was estimated by partial η^2^-values [59-61]. To assess the relative effects of family and population on the pheromone composition, the η^2^-values for both effects were compared over all peaks in paired t-tests after checking for normal distributions using Kolmogorov-Smirnov tests. All tests were computed using SPSS 13.0 software.

#### Association between geographical and chemical distance

The geographical distances between all sampled populations were calculated from the population coordinates with the DIVA-GIS software [62] and subsequently log-transformed. The chemical distances between the populations were calculated as follows: The mean for each of the 20 Aitchison-transformed pheromone components was calculated for all populations. The chemical distance between two given populations x and y was calculated as the Euclidean distance according to the formula Dchem(x,y)=∑i=1n(xi−yi)2
 MathType@MTEF@5@5@+=feaafiart1ev1aaatCvAUfKttLearuWrP9MDH5MBPbIqV92AaeXatLxBI9gBaebbnrfifHhDYfgasaacH8akY=wiFfYdH8Gipec8Eeeu0xXdbba9frFj0=OqFfea0dXdd9vqai=hGuQ8kuc9pgc9s8qqaq=dirpe0xb9q8qiLsFr0=vr0=vr0dc8meaabaqaciaacaGaaeqabaqabeGadaaakeaacqWGebardaWgaaWcbaGaem4yamMaemiAaGMaemyzauMaemyBa0gabeaakiabcIcaOiabdIha4jabcYcaSiabdMha5jabcMcaPiabg2da9maakaaabaWaaabCaeaacqGGOaakcqWG4baEdaWgaaWcbaGaemyAaKgabeaakiabgkHiTiabdMha5naaBaaaleaacqWGPbqAaeqaaOGaeiykaKYaaWbaaSqabeaacqaIYaGmaaaabaGaemyAaKMaeyypa0JaeGymaedabaGaemOBa4ganiabggHiLdaaleqaaaaa@4AB9@ with x_i _as the mean of pheromone component i of population x. To be able to combine data sets 1 and 2, which differed slightly due to the differences in GC-MS set-ups, chemical distances were normalized. To this end, we assumed that the chemical distances between two populations that were sampled in both data sets were identical and served as a reference. Consequently, we were able to normalize the chemical distances of the two data sets with the distance between Würzburg and Schweinfurt, Würzburg and Düsseldorf, and Schweinfurt and Düsseldorf, respectively. The respective chemical distance was set to 1 in both data sets and all other values were converted to relative chemical distances. The normalizations based on the three different reference distances revealed qualitatively the same results in the following analyses, indicating that the procedure yielded valid results. The relationship between geographical and chemical distances was visualized using a scatter-plot and a linear regression line. We tested for a correlation between the matrices of geographical and chemical distances using a Mantel test that can deal with missing values using the software R 2.3.0 (mantel.test from the ncf package) [63,64]. P-values were calculated based on 100,000 resamplings. Mantel tests were performed with each dataset separately, and with the normalized combined dataset. All tests were conducted with both C_25_- and C_25_/C_27_-type males and with C_25_-type-males only to exclude effects of different relative frequencies of both chemo-types across populations.

## Competing interests

The author(s) declares that there are no competing interests.

## Authors' contributions

All authors participated in the design of the study. MK and JK collected the specimens in Würzburg, Schweinfurt and Italy, they designed the GC-MS experiments, performed the statistical analysis and drafted the manuscript. ES conceived of the study, participated in the statistical analysis and interpretation of the data and critically revised the manuscript. All authors read and approved the final manuscript.
